# New Gait Representation Maps for Enhanced Recognition in Clinical Gait Analysis

**DOI:** 10.3390/bioengineering12101130

**Published:** 2025-10-21

**Authors:** Nagwan Abdel Samee, Mohammed A. Al-masni, Eman N. Marzban, Abobakr Khalil Al-Shamiri, Mugahed A. Al-antari, Maali Ibrahim Alabdulhafith, Noha F. Mahmoud, Yasser M. Kadah

**Affiliations:** 1 Department of Information Technology, College of Computer and Information Sciences, Princess Nourah bint Abdulrahman University, Riyadh 11671, Saudi Arabia; nmabdelsamee@pnu.edu.sa (N.A.S.); mialabdulhafith@pnu.edu.sa (M.I.A.); 2 Department of Artificial Intelligence and Data Science, College of Artificial Intelligence Convergence, Sejong University, Seoul 05006, Republic of Korea; en.mualshz@sejong.ac.kr; 3 Biomedical Engineering Department, Cairo University, Giza 12613, Egypt; eman.marzban@eng1.cu.edu.eg; 4 School of Computer Science, University of Southampton Malaysia, Iskandar Puteri 79100, Johor, Malaysia; a.k.n.al-shamiri@soton.ac.uk; 5 Rehabilitation Sciences Department, Health and Rehabilitation Sciences College, Princess Nourah bint Abdulrahman University, Riyadh 11671, Saudi Arabia; nfmahmoud@pnu.edu.sa; 6 Electrical and Computer Engineering Department, King Abdulaziz University, Jeddah 22254, Saudi Arabia; ykadah@kau.edu.sa

**Keywords:** gait analysis, gait impairment, gait pathology classification, deep learning, gait cycle maps

## Abstract

Gait analysis is essential in the evaluation of neuromuscular and musculoskeletal disorders; however, traditional approaches based on expert visual observation remain subjective and often lack consistency. Accurate and objective assessment of gait impairments is critical for early diagnosis, monitoring rehabilitation progress, and guiding clinical decision-making. Although Gait Energy Images (GEI) have become widely used in automated, vision-based gait analysis, they are limited in capturing boundary details and time-resolved motion dynamics, both critical for robust clinical interpretation. To overcome these limitations, we introduce four novel gait representation maps: the time-coded gait boundary image (tGBI), color-coded GEI (cGEI), time-coded gait delta image (tGDI), and color-coded boundary-to-image transform (cBIT). These representations are specifically designed to embed spatial, temporal, and boundary-specific features of the gait cycle, and are constructed from binary silhouette sequences through straightforward yet effective transformations that preserve key structural and dynamic information. Experiments on the INIT GAIT dataset demonstrate that the proposed representations consistently outperform the conventional GEI across multiple machine learning models and classification tasks involving different numbers of gait impairment categories (four and six classes). These findings highlight the potential of the proposed approaches to enhance the accuracy and reliability of automated clinical gait analysis.

## 1. Introduction

Gait, or land human locomotion, is a complex movement determined by the coordinated actions of the skeletal, muscular, and nervous systems. Various factors, such as gender, age, pathological conditions, and muscle fatigue, can influence gait, often resulting in abnormal walking patterns that impair mobility and overall functionality [1,2]. These abnormalities may affect both the upper and lower limbs, making clinical gait analysis a valuable tool for assessing neurological and physical disorders, including stroke, multiple sclerosis, and Parkinson’s disease. For example, hemiplegic gait, characterized by spasticity and imbalance, is frequently observed in stroke survivors [3]. Assessing the severity of this dysfunction provides critical insights into a patient’s recovery progress.

Although gait abnormalities can often be visually observed under uncontrolled conditions, the interpretation of individual gait patterns traditionally relies on rehabilitation experts who use visual observation to evaluate walking characteristics. However, this approach is subjective and highly dependent on the evaluator’s expertise [4]. Physicians typically rely on quantitative parameters, such as spatio-temporal, kinetic, kinematic, and physiological or anthropometric measures, to assess gait-related pathologies during clinical gait analysis [5].

Automated gait analysis systems have emerged as a promising alternative, offering precise identification of gait impairments, enabling early diagnosis, and supporting informed clinical decision-making. Moreover, automated gait analysis has garnered significant attention in fields such as healthcare, rehabilitation, sports performance monitoring, and security surveillance, providing a more objective and consistent assessment [6].

By analyzing biomechanical gait features such as speed, step length, and stance and swing times, these systems can detect impairments and even differentiate between disorders based on severity. Furthermore, such analyses can predict fall risks in older adults and assess head impact risks in athletes [2,7], highlighting their potential for broader applications.

Over the past two decades, there has been increasing interest in clinical applications of gait assessment. Methods for gait impairment analysis can be broadly categorized based on sensing modalities into motion and biosignal measurements (e.g., wearable sensors such as accelerometers, gyroscopes, Electromyography (EMG), and Electroencephalography (EEG)) and vision-based measurements (e.g., non-wearable sensors such as 2D and 3D cameras) [1]. Among these, vision-based approaches have gained popularity due to their ability to seamlessly integrate with computer vision and artificial intelligence systems for automated gait analysis. For instance, spatial features can be learned through model training using image data, such as frames captured from video clips of individuals walking. In some studies, even signal-based modalities are converted into images to facilitate gait pattern recognition. Examples include spectrogram images generated from acceleration signals [8] and recurrence plot images derived from vertical Ground Reaction Force (vGRF) signals [9].

A foundational concept in vision-based modalities is the use of gait silhouette images, which require a carefully designed environment to record and extract these silhouettes from video footage of a patient’s movement. However, using individual silhouette images alone is ineffective, as they lack the temporal information necessary to represent a complete gait pattern. Instead, combining a sequence of consecutive silhouette images to represent an entire gait cycle (i.e., comprising stance and swing phases) is critical for enabling machine learning models to effectively identify gait impairments.

A well-known method for generating such a combination is the Gait Energy Image (GEI) [10,11,12]. GEI is generated by averaging a sequence of aligned gait silhouette images over a complete gait cycle, producing a single composite image that captures the spatial and temporal features of the gait cycle. While GEI is widely used as an input for machine learning models in gait analysis, it has notable limitations. Specifically, GEI does not account for boundary information or time-resolved dynamics, resulting in a grayscale image that represents the subject’s motion through the temporal mean intensity of each pixel. Additionally, GEI may fail to preserve fine-grained motion details, which could limit its utility in identifying subtle gait abnormalities.

In contrast, this study introduces four new gait representation maps designed to incorporate richer and more clinically relevant information compared to GEI. The first proposed method is the time-coded gait boundary image (tGBI), which accumulates the boundaries of binary silhouette images across the gait cycle rather than the silhouettes themselves, thereby preserving boundary-specific information. The second is the color-coded GEI (cGEI), which divides the gait cycle into three segments and assigns each segment to a distinct color channel, creating a time-resolved and color-enhanced representation. The third image representation, the time-coded gait delta image (tGDI), emphasizes motion dynamics by capturing the differences between consecutive images in the gait sequence, with indices reflecting their position within the gait cycle. Finally, the color-coded boundary-to-image transform (cBIT) encodes the boundary points of each binary silhouette as distinct lines within a color image, offering a novel visualization of boundary transitions.

These proposed representations address the limitations of GEI and provide a more comprehensive approach to clinical gait analysis by preserving detailed motion dynamics and incorporating time-resolved information.

The main contributions of this paper can be summarized as follows. First, we propose four new gait image representations, each designed to emphasize distinct dynamic features of the gait cycle, including boundary information, motion dynamics, and time-resolved characteristics. Second, we conduct gait impairment recognition experiments and demonstrate how these new representation maps achieve superior performance across various machine learning approaches when compared to the conventional GEI. Third, we evaluate the proposed representations using datasets comprising two different numbers of gait impairment classes (four and six), encompassing normal gait, severe gait impairment, and impairments affecting the left and right legs and arms.

## 2. Related Works

Gait representation plays a crucial role in recognizing and analyzing human gait patterns, especially in clinical settings. Traditional methods have been foundational, but with advancements in technology, newer techniques have emerged to better capture the temporal and spatial dynamics of human motion.

One of the most widely used traditional methods for gait recognition is the GEI, which creates a single image by averaging silhouette frames over the entire gait cycle. GEI captures the overall shape and motion of an individual’s gait [12]. The advantage of GEI lies in its simplicity and its ability to encapsulate overall gait dynamics. It has been a foundational technique in human identification and gait analysis for detecting pathologies. However, GEI’s reliance on silhouette images can lead to the loss of finer details, such as boundary variations and subtle motion characteristics, which are essential for accurately recognizing gait abnormalities in clinical settings [6,12].

Over time, various techniques have been developed to address the limitations of GEI, enhancing the representation of gait patterns in more comprehensive and clinically relevant ways [13,14,15,16,17]. Recurrence plot images derived from signals like vertical Ground Reaction Force (vGRF) offer one such advancement. Recurrence plots visualize the temporal patterns in gait signals, providing a more nuanced view of the gait cycle that is particularly valuable for clinical assessment. Studies have demonstrated that using recurrence plots can improve the accuracy of gait classification by capturing subtle gait abnormalities that may be overlooked by GEI [9].

In addition, the use of spectrogram images derived from accelerometer data has shown potential in improving the recognition of pathological gait. Spectrograms transform raw acceleration signals into time-frequency representations, providing a richer and more comprehensive view of gait dynamics, which is critical for detecting abnormalities linked to conditions such as Parkinson’s disease [8,18,19]. For instance, El-Ziaat et al. [8] proposed a multi-feature fusion approach to enhance the detection of freezing of Gait episodes in patients with Parkinson’s disease. The study combined time-domain statistical feature engineering with spectrogram-based time-frequency analysis, leveraging Convolutional Neural Networks (CNNs) to extract deep features from spectrogram images. Linda et al. [20] introduced a Color-Mapped Contour Gait Image (CCGI) technique to enhance Cross-View Gait Recognition (CVGR) using deep convolutional neural networks. CCGI addressed challenges associated with cross-view variations by encoding spatial and temporal information through color mapping, resulting in regularized contour images with fewer outliers.

Machine learning and deep learning techniques have greatly expanded the capabilities of gait recognition, enabling systems to handle large datasets and automatically extract relevant features from raw gait data. These methods are particularly advantageous in clinical applications, where the accuracy and efficiency of gait classification are paramount.

Traditional machine learning approaches, such as K-Nearest Neighbors (KNN), Linear Discriminant Analysis (LDA), and Neural Networks (NN), have been used for gait classification [21,22,23]. For instance, Zhang et al. [22] developed a gait recognition framework based on a Siamese neural network to extract gait features for human identification. The network employed distance metric learning to optimize similarity metrics for distinguishing between gaits of the same and different individuals. The study employed GEIs as input and utilized the KNN algorithm for classification.

The advent of deep learning, particularly CNNs, has revolutionized gait recognition by enabling automatic feature extraction from raw gait data. CNNs excel in image-based gait recognition tasks, where they can learn to detect important spatial and temporal features in gait images, such as GEIs. Several studies have successfully applied CNNs to recognize pathological gait patterns with high accuracy, highlighting the promise of deep learning in this field [24,25,26,27,28,29,30,31,32,33]. Castro et al. [24] proposed a gait identification framework leveraging CNNs to extract high-level features from low-level optical flow motion data. Their approach introduced a preprocessing pipeline to organize and normalize motion features as input for the CNN, which was designed to generate discriminative gait signatures. In [25], the authors presented a gait recognition system using CNNs and optical flow for feature extraction. The system incorporated two key modules: motion detection and tracking, and feature extraction. Motion detection employed background subtraction to isolate and segment the moving area, focusing on the spatial silhouette. The feature extraction module calculated gait cycles from silhouette sequences and constructed GEI. Optical flow is applied to GEIs to isolate dynamic components, excluding static parts. These processed features were input into a CNN to learn unique gait patterns. Al-masni et al. [28] proposed an innovative method for analyzing gait impairments by introducing silhouette sinograms, a one-dimensional representation that captures the relative variations in motion from silhouette images. These sinograms are created by calculating the distance and angle between the centroid and boundary points of detected silhouettes, effectively highlighting motion patterns. They utilized a one-dimensional convolutional neural network (1D CNN) to process consecutive silhouette sinogram signals, effectively learning spatiotemporal features through assisted knowledge learning.

In recent years, researchers have explored the integration of silhouette extraction and gait recognition within unified frameworks to address the limitations of traditional stepwise methods. By combining key processes like segmentation, feature extraction, and classification into a single learning framework, these approaches offer enhanced performance and efficiency. For instance, Nahar et al. [34] proposed a unified CNN for gait recognition that merged feature extraction and recognition into an end-to-end framework. Their method emphasized dynamic areas of gait using gait entropy images as input, which captured motion information while being robust to covariate conditions such as varying viewpoints and walking styles. Similarly, Song et al. [35] introduced GaitNet, an end-to-end network designed for gait-based human identification. This framework integrated silhouette segmentation and classification through two jointly trained CNNs. The joint learning approach overcame inefficiencies of traditional modular pipelines by adjusting each component to optimize the overall objective. The results of both studies highlighted the potential of end-to-end frameworks to enhance gait recognition performance by eliminating the limitations of fixed, independent modules and enabling more adaptive, streamlined learning.

## 3. Materials and Methods

### 3.1. Dataset

In this study, we utilized the publicly available INIT Gait Database (https://www.vision.uji.es/gaitDB/ (accessed 20 March 2025)), which provides high-quality binary silhouette sequences extracted from RGB video recordings. These recordings were captured at LABCOM, a specialized audiovisual studio at the University Jaume I. The dataset was created under controlled conditions with uniform green background and a lateral view camera setup, ensuring precise silhouette extraction.

The dataset includes ten healthy volunteers (nine males and one female), who were instructed to simulate a variety of gait patterns. These patterns were designed to resemble pathological gait abnormalities typically observed in clinical practice. The dataset comprises eight gait types: natural normal walking (NM), abnormal right leg (RL), abnormal left leg (LL), abnormal full body (FB), abnormal right arm (RA), abnormal left arm (LA), and partial arm impairments in which the swing of the left or right arm is restricted to approximately half of its normal range (LA-0.5 and RA-0.5). Normal walking represents the unaffected gait of a healthy subject, whereas the abnormal categories capture disturbances such as shortened stride length, reduced or absent arm swing, and shuffling gait with bent knees and small steps. Together, these categories provide a clinically inspired range of gait patterns, which in our study are treated as separate classes for multiclass recognition rather than a simple binary normal/abnormal classification.

Each participant performed two sequences per gait style, resulting in a total of 160 recorded sequences (10 subjects × 8 gait styles × 2 sequences). Each recording contained a variable number of gait cycles, depending on the simulated impairment. For example, participants walking with full-body impairment (FB) moved more slowly, producing a greater number of cycles within a single sequence compared to normal walking. From these sequences, the number of gait cycles extracted for each style were: FB (112), LL (102), RL (113), NM (85), LA (90), and RA (85). In total, all 160 recorded sequences contain 41,104 silhouette images, with each frame having a resolution of 400 × 800 pixels.

To maintain subject independence and address gender distribution, data were split at the subject level: 8 males (80%) for training and 2 subjects (20%; one male, one female) for testing. This setup ensured that the female participant was excluded from training but included in testing, thereby allowing evaluation of generalization across gender. The proposed representations maintained robust performance even on female samples in the test set. It should be noted that the ten participants varied in height and weight, although no obese individuals were included. This natural variation adds diversity to the dataset, but without introducing extreme body-size differences that could confound silhouette-based gait analysis.

This dataset serves as a valuable resource for studying gait variations and evaluating automated gait classification models.

### 3.2. Proposed Framework

In this study, a novel gait representation framework is introduced to enhance the detection of gait impairments. The key innovation of this work lies in the generation of new gait representation images, which are designed to capture distinct dynamic features of the gait cycle, including boundary information, motion dynamics, and time-resolved characteristics. These four newly developed gait representations provide a richer feature extraction process compared to the conventional GEI, leading to superior classification performance across different machine learning approaches.

To effectively utilize these new gait representations, a hybrid deep learning and Principal Component Analysis (PCA) approach is employed. The proposed Computer-Aided Diagnosis (CAD) system follows a structured three-stage process. First, the gait representation preparation stage involves constructing these four distinct gait image representations to emphasize key movement characteristics. Next, deep feature extraction is performed using pretrained convolutional neural networks (CNNs) including AlexNet which operate within a transfer learning framework to extract high-level deep features while mitigating overfitting risks. Finally, in the dimensionality reduction and classification stage, PCA is applied to refine the extracted deep features by selecting the most significant principal components, addressing multicollinearity while preserving essential variance. This step ensures a more compact and efficient feature space, improving the overall classification accuracy.

The proposed CAD system is structured into four main modules. The first module, Gait Image Generation, involves constructing the new gait representations that highlight critical movement characteristics. The second module, Feature Extraction, leverages pretrained CNNs to obtain discriminative deep features from the generated images. The third module, Dimensionality Reduction, applies PCA to optimize feature selection and eliminate redundancy in the extracted features. Lastly, the Classification and Performance Evaluation module involves implementing traditional machine learning classifiers to categorize gait impairments and assess performance using standard CAD evaluation metrics.

**Figure 1:**
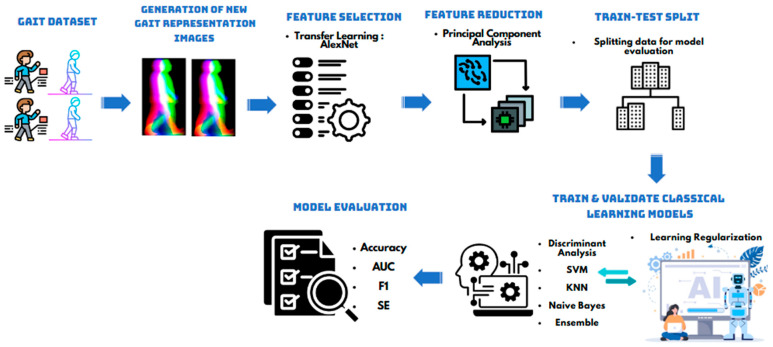
Schematic of the proposed framework: gait map generation, feature extraction using AlexNet, PCA-based dimensionality reduction, train-validation-test splitting, training and validation of classical classifiers, and final model evaluation.

To validate the effectiveness of the proposed framework, gait impairment recognition experiments were conducted on datasets consisting of four-class and six-class gait impairments, encompassing normal gait, severe gait impairments, and impairments affecting the left and right legs and arms. A schematic of the proposed framework is presented in Figure 1.

#### 3.2.1. Generation of New Gait Representation Images

In this section, four new gait representation images are proposed as alternatives to the gait energy image (GEI) representation. The proposed representations are designed to incorporate additional or more clinically relevant information than GEI provides. Three of the new representations are intended for both human and machine interpretation, while the fourth is primarily designed for machine interpretation. All representations begin with preprocessing steps to align and standardize the size of binary silhouettes, followed by the extraction of their boundaries.

##### Binary Silhouettes Preprocessing

A. Image Cropping:

The first step is to segment and crop all binary silhouettes in the gait cycle of each subject, so they fit within the same image size. This is achieved by projecting the original silhouettes along the horizontal and vertical axes, then identifying the start and end points as the minimum and maximum positions of nonzero elements. Given an initial binary silhouette image n in the sequence $S_{n}(x,y)$ , the procedure can be expressed mathematically as follows:

(1) $$S_{nx}\left(y\right)=\sum_{x}S(x,y)\mathrm{and}\;S_{ny}\left(x\right)=\sum_{y}S(x,y).$$

The cropped image $S_{n}^{c}\left(x,y\right)$ is subsequently derived as the subset of rows and columns for which the projections in both the x and y directions are nonzero such that,

(2) $$S_{n}^{c}\left(x,y\right)={\left\{S_{n}\left(x,y\right)\right\}}_{{\;S}_{nx}\left(y\right)>0\;and{\;S}_{ny}\left(x\right)>0}\;.$$

The cropped image sizes $N_{nx}$ and $N_{ny}$ are obtained as the cardinality of all nonzero elements in the projection vectors $S_{ny}\left(x\right)$ and $S_{nx}\left(y\right)$ , which can be calculated as the 0-norm of each:

(3) $$N_{nx}={\left\|S_{ny}\left(x\right)\right\|}_{0}\;,N_{ny}={\left\|S_{nx}\left(y\right)\right\|}_{0}\;.$$

Each binary silhouette is cropped in both the horizontal and vertical directions to retain only the points between the start and end locations. This results in a sequence of binary silhouette images of variable horizontal and vertical dimensions. To standardize their sizes, all images are zero-padded equally from both directions to match the maximum height and width found among cropped images, resulting in uniformly sized binary silhouette images. The common binary silhouette sizes in a particular sequence are obtained as follows:

(4) $$N_{x}=\;\underset{n}{\max}\left\{N_{nx}\right\}\;,\;{\;N}_{y}=\;\underset{n}{\max}\left\{N_{ny}\right\}.$$

The result of zero-padding all binary silhouette images in the sequence to the common sizes $N_{x}$ and $N_{y}$ is termed $S_{n}^{cz}\left(x,y\right)$ for the image n in the sequence. This will ensure consistency of subsequent movement assessment steps for the different silhouettes in the gait sequence.

B. Centroid and Boundary Calculations:

For each gait sequence, the centroid location $\left(c_{nx},c_{ny}\right)$ of every uniformly sized, cropped binary silhouette image $S_{n}^{cz}\left(x,y\right)$ is calculated as the center of mass of the silhouette region, assuming uniform mass density throughout. This can be expressed mathematically as:

(5) $$c_{nx}=\;\frac{\sum_{y}\sum_{x}x\;S_{n}^{cz}\left(x,y\right)}{\sum_{y}\sum_{x}\;S_{n}^{cz}\left(x,y\right)}\;,\;c_{ny}=\;\frac{\sum_{y}\sum_{x}y\;S_{n}^{cz}\left(x,y\right)}{\sum_{y}\sum_{x}\;S_{n}^{cz}\left(x,y\right)}.$$

**Figure 2:**
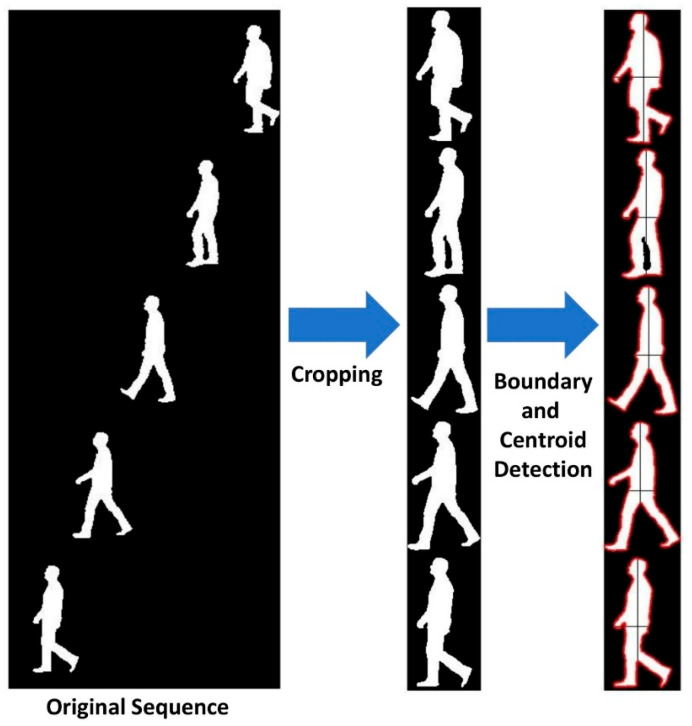
Example of cropping process and boundary detection on a gait silhouette sequence.

Next, the uniformly sized, cropped binary silhouette images undergo a boundary tracing operation to obtain an ordered sequence of points that represent the silhouette boundary. This approach is more sophisticated than standard edge detection, as it provides an ordered sequence of boundary points rather than a simple binary contour image —an important advantage when dealing with complex silhouette shapes. Several existing methods can perform boundary tracing, such as pixel-following or vertex-following methods, which typically assume that the input binary images are zero-padded with no boundary points at the image matrix edges. In this study, the boundary tracing procedure was implemented using the Moore-Neighbor tracing algorithm, modified with the Jacob’s stopping criteria [36]. The process begins at a boundary point, typically the uppermost-leftmost pixel, and examines its eight neighboring pixels one by one in the same direction (e.g., clockwise). Starting from a neighbor with a value of zero (background), it selects the first neighbor with a value of one (part of the silhouette) as the next point in the boundary sequence. This procedure is repeated until one of Jacob’s stopping criteria is satisfied, indicating that the boundary sequence has returned to the starting point. The result of this boundary detection operation is a list of boundary points’ horizontal and vertical coordinates, $\left\{{{\vec{b}}_{nx},\vec{b}}_{ny}\right\}$ , for each cropped binary silhouette image $S_{n}^{cz}\left(x,y\right)$ . It should be noted that the size of these boundary point sequences may vary across different images within the same gait sequence. Figure 2 depicts an example of the boundary and centroid detection.

C. Gait Cycle Detection:

Accurate detection of the gait cycle is a critical step in gait analysis, as it ensures that the extracted features correspond to a complete and consistent motion sequence. A gait cycle is defined as the time interval between two consecutive occurrences of a specific event in the walking pattern, such as heel strike or toe-off of the same foot. Detecting the gait cycle involves analyzing the temporal patterns in a sequence of gait frames and identifying key phases of the walking motion. Gait cycle detection typically relies on the analysis of temporal variations in the subject’s motion, often represented by binary silhouettes or joint trajectories. Methods such as motion energy profiles, centroid displacement, or periodicity analysis are commonly employed. Motion energy profiles, derived from the horizontal or vertical projection of silhouette pixels, are particularly effective in identifying repetitive patterns associated with walking. The detection process involves identifying the starting and ending frames of a complete gait cycle. This is achieved by locating periodic peaks or troughs in the motion energy profile that correspond to significant events, such as the maximum extension of a leg or the alignment of both feet. In this work, the detection was based on the calculation of 2D correlation coefficients across all cropped binary silhouette images $S_{n}^{cz}\left(x,y\right)$ in the gait sequence or their Fourier transformation magnitudes. The rationale for this selection lies in the fact that the 2D correlation coefficient is a robust statistical measure for assessing similarity between binary silhouettes, with inherent properties that make it particularly advantageous in scenarios where noise, such as segmentation errors, may interfere with data analysis. This results in two signals with the 2D correlation coefficients vs. time for images ( $Corr_{I}\left(n\right)$ ) or Fourier transform magnitudes ( $Corr_{F}\left(n\right)$ ) that are expected to have periodic peaks and valleys that can be used to robustly detect the gait cycle. Based on practical experimentation, the valleys were found to be more reliable reference points in each cycle due to their clearly defined locations, whereas the peaks tend to be more difficult to locate because of flatter regions observed in certain gait cycles.

**Figure 3:**
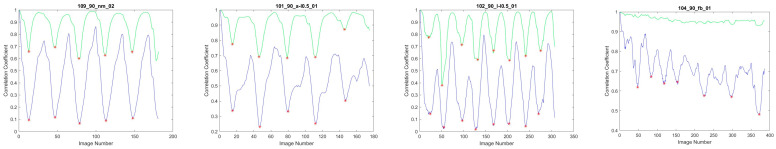
Gait cycle detection illustrated through correlation coefficient curves, showing from left to right the valley detection results for normal gait, arm and leg impairments, and full-body abnormalities. Blue curves represent image correlation coefficients, green curves indicate frequency correlations, and stars mark local minima corresponding to the start or end of new gait cycles.

The valley detection algorithm employed in this study is a simple minima detection method that considers the prominence of a local minimum. Prominence is a measure of how the valley compares with respect to its depth and location relative to other valleys. To compute the prominence of a valley, the closest points before and after the detected valley with the same correlation coefficient value are first identified. Each of these outer points can be either a point on the correlation coefficient curve or the end of data. Then, we find the highest correlation coefficient peaks in both the left and right intervals between the detected valley and the two outer points. The difference in correlation coefficient values between the smaller of these two peaks and the detected valley is taken as a measure of the valley prominence. To ensure reliable detection of valleys, a minimum prominence threshold of 0.1, corresponding to 10% of the range of correlation coefficient values in each signal, was used in this study. Figure 3 illustrates an example of a correlation curve used to identify gait cycles.

##### Gait Sequence Image Representations

A. Gait Energy Image (GEI):

The GEI is a widely used spatiotemporal representation in gait analysis, which summarizes the motion information of a subject over a complete gait cycle into a single grayscale image. The GEI is computed from a sequence of binary silhouette images, where each silhouette represents the subject’s body in a single frame of the gait cycle. To ensure consistency across frames, all binary silhouettes are preprocessed to align and normalize the subject’s position and size. The alignment process centers the silhouettes by translating each frame so that the centroid of the silhouette coincides with a fixed reference point.

Additionally, the silhouettes are scaled to a uniform height and width to account for variations in subject size or camera distance.

In this work, the GEI representation is calculated as the average of the aligned binary silhouette images over a complete gait cycle. Given a sequence of N cropped and aligned binary silhouettes $S_{n}^{cz}\left(x,y\right)$ , where n ∈ {1, 2, …, N} and $\left(x,y\right)$ denotes the pixel coordinates, the GEI is computed as:

(6) $$GEI\left(x,\;y\right)=\frac{1}{N}\;\sum_{n=1}^{N}\;S_{n}^{cz}\left(x,y\right).$$

**Figure 4:**
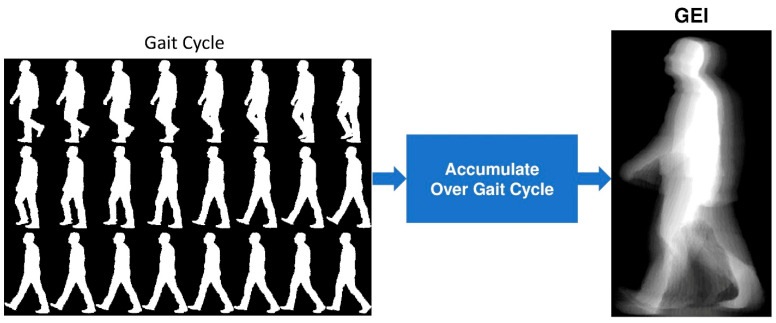
Generation process of the Gait Energy Image (GEI) map from a sequence of silhouette images.

Each pixel value in the resulting GEI represents the temporal mean intensity of that pixel across the gait cycle, providing a compact and informative representation of the subject’s motion. So, the resulting GEI is a single grayscale image summarizing the gait motion cycle. Figure 4 shows an example of a generated GEI.

**Figure 5:**
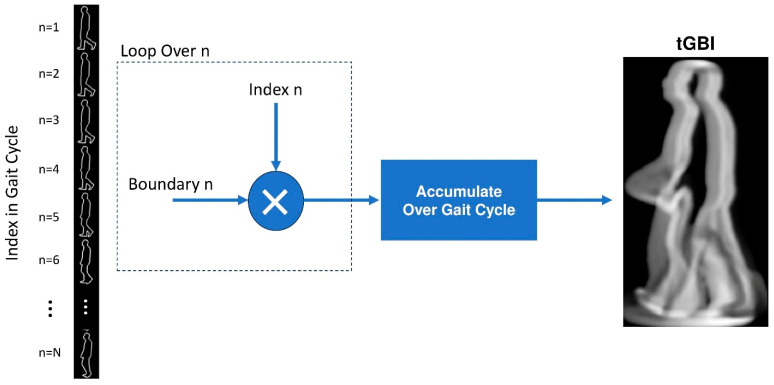
Generation process of the time-coded Gait Boundary Image (tGBI) from binary silhouette boundaries.

B. Time-Coded Gait Boundary Image (tGBI):

In this proposed representation, instead of accumulating the binary silhouettes across images in a gait cycle, the boundaries of the binary silhouettes are accumulated. Additionally, each boundary is weighed according to its time index within the gait cycle. Consequently, this representation is referred to as the Time-Coded Gait Boundary Image (GBI). The motivation for this new representation is to emphasize the importance and visibility of the boundary changes throughout the gait cycle, without interference or even obstruction from the filled regions within the binary silhouettes. As a result, this representation has the potential to enhance both human and machine interpretation of gait patterns. An example of a generated tGBI is shown in Figure 5.

The calculation of the tGBI representation proceeds as follows. First, the boundary detection operation produces a list of boundary point coordinates, $\left\{{{\vec{b}}_{nx},\vec{b}}_{ny}\right\}$ , for each cropped binary silhouette image $S_{n}^{cz}\left(x,y\right)$ . A new boundary image $B_{n}^{cz}\left(x,y\right)$ , having the same size as $S_{n}^{cz}\left(x,y\right)$ is then created for each binary silhouette, defined as:

(7) $$B_{n}^{cz}\left(x,y\right)=\left\{\begin{array}{cc} 1\;, & \;(x,y)\in \;\left\{{{\vec{b}}_{nx},\;\vec{b}}_{ny}\right\}\\ 0\;, & Otherwise \end{array}\right.\;.$$

Subsequently, the tGBI representation is computed by accumulating the boundary images of all binary silhouettes in the gait sequence, weighed by their respective time indices, as follows:

(8) $$tGBI\left(x,\;y\right)=\frac{1}{N}\;\sum_{n=1}^{N}\;{n\;\cdot B}_{n}^{cz}\left(x,y\right).$$

**Figure 6:**
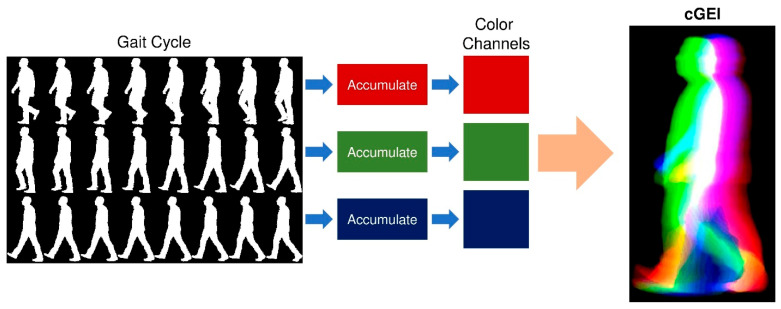
Generation of the color-coded Gait Energy Image (cGEI) by segmenting the gait cycle into temporal color channels.

C. Color-Coded Gait Energy Image (cGEI):

This proposed representation is a variant of the traditional GEI that incorporates color coding for time in the gait cycle. In particular, the gait cycle is divided into three temporal segments, and a partial GEI is computed for each. These partial images are then assigned to the red, green, and blue colors, respectively, to form the Color Gait Energy Image (cGEI). An example of a generated cGEI is illustrated in Figure 6. This representation provides a time-resolved view of the gait sequence, offering an advantage over the conventional GEI, which lacks time resolving capability. As a result, the cGEI is suitable for both human and machine interpretation. Mathematically, the cGEI representation can be defined as a color image with three color components that are expressed as follows:

(9) $${cGEI\left(x,\;y\right)}_{Red}=\frac{1}{N}\;\sum_{n=1}^{\frac{N}{3}}\;S_{n}^{cz}\left(x,y\right).$$

(10) $${cGEI\left(x,\;y\right)}_{Green}=\frac{1}{N}\;\sum_{n=\frac{N}{3}+1}^{\frac{2N}{3}}\;S_{n}^{cz}\left(x,y\right).$$

(11) $${cGEI\left(x,\;y\right)}_{Blue}=\frac{1}{N}\;\sum_{n=\frac{2N}{3}+1}^{N}\;S_{n}^{cz}\left(x,y\right).$$

**Figure 7:**
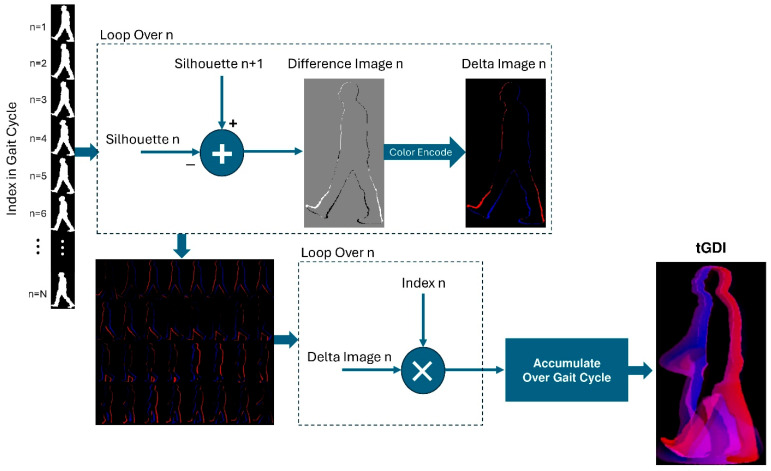
Generation of the time-coded Gait Delta Image (tGDI) highlighting motion changes between consecutive frames.

D. Time-Coded Gait Delta Image (tGDI):

This proposed representation is designed to highlight the differences between consecutive images in the gate sequence as representatives of the gait motion along with their indices within the gate cycle. To construct this representation, each image in the gate sequence is subtracted from its preceding image, producing delta images with positive, negative, and zero values. The positive and negative parts indicate regions where motion occurred either to (positive values) or from (negative values) them. Two simple thresholding masks are applied to the difference image (i.e., delta image) to generate separate maps of positive and negative values. These maps are then multiplied by their respective time indices within the gait cycle. Finally, the resulting time-weighted positive and negative maps are concatenated as the red and blue channels, respectively, to form the Time-Coded Gait Delta Image (tGDI). This representation allows a better and finer time-resolved view of the sequence of images in the gait cycle. It offers the added value of having the time variable as well as the movement of different parts information present in the final image. Owing to its intuitive concept and straightforward computational procedure, the tGDI is suitable for both human and machine interpretation. An example of a generated tGDI is presented in Figure 7. To compute the tGDI representation, each cropped binary silhouette image $S_{n}^{cz}\left(x,y\right)$ is subtracted from the subsequent image $S_{n+1}^{cz}\left(x,y\right)$ to create a delta image $d_{n}^{cz}\left(x,y\right)$ of the same size as $S_{n}^{cz}\left(x,y\right)$ . This delta image contains positive and negative values, depending on how the gait motion went to or from different parts of the image. A new color delta image $D_{n}^{cz}\left(x,y\right)$ is then created from each delta image whereby the red channel has the positive values of the delta image, while the blue channel has the absolute values of the negative values. This process can be mathematically expressed as follows:

(12) $${D_{n}^{cz}\left(x,y\right)}_{Red}=\left\{\begin{array}{c} 1\;,\;d_{n}^{cz}\left(x,y\right)>0\;\\ 0\;,\;\;\;\;\;\;Otherwise \end{array}\right.\;.$$

(13) $${D_{n}^{cz}\left(x,y\right)}_{Blue}=\left\{\begin{array}{c} 1\;,\;d_{n}^{cz}\left(x,y\right)<0\;\\ 0\;,\;\;\;\;\;\;Otherwise \end{array}\right.\;$$

The tGDI representation is obtained by accumulating the color delta images of all binary silhouettes in the gait sequence, weighed by their respective time indices, as follows:

(14) $$tGDI\left(x,\;y\right)=\frac{1}{N}\;\sum_{n=1}^{N}\;{n\;\cdot D}_{n}^{cz}\left(x,y\right)$$

**Figure 8:**
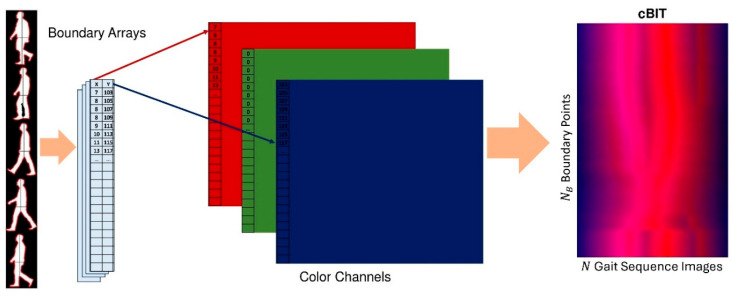
Generation process of the color-coded Boundary-to-Image Transform (cBIT) using boundary points of silhouette images.

E. Color-Coded Boundary-to-Image Transform (cBIT):

This proposed representation adopts a different approach by encoding the complete information of a gait sequence into a single image. The basic idea is to encode the boundary points of each binary silhouette as a single line in a color image representation such that the x -coordinates are written as the red color information, while the y -coordinates are written as the blue-color information for that line. In this way, the image lines collectively contain the full boundary information of all binary silhouettes in the gait sequence. In principle, this information enables the reconstruction of the original binary silhouettes by recovering the boundary points from the encoded image and applying a simple flood fill algorithm. As such, this representation can be considered as a unique transformation with a one-to-one correspondence between its two domains, and is thus referred to as the Color-Coded Boundary-to-Image Transform (cBIT) representation. Even though the information in the resultant image encompasses all the information in the original sequence, it is very difficult to interpret by humans given its unintuitive appearance that does not resemble gait images. Rather, it is intended for such techniques as deep learning whereby they can be designed and trained to take advantage of the full scope of the embedded information in that representation. Hence, this representation can be labelled as suitable for machine interpretation only. An example of a generated cBIT is shown in Figure 8.

The calculation of the cBIT representation begins with the result of the boundary detection operation, obtained as a list of horizontal and vertical locations of boundary points, $\left\{{{\vec{b}}_{nx},\vec{b}}_{ny}\right\}$ , for each cropped binary silhouette image $S_{n}^{cz}\left(x,y\right)$ . The size of all boundary point arrays from all images in the gait sequence is selected as their maximum, $N_{B}$ . To ensure uniformity, all boundary point arrays are resampled to this common size, if necessary, resulting in $\left\{{{\vec{b'}}_{nx},\vec{b'}}_{ny}\right\}$ . Subsequently, the cBIT image $T\left(x,y\right)$ of size $N_{B}\times N$ is constructed for all binary silhouettes as follows:

(15) $${T\left(x,n\right)}_{Red}\;=\;{\vec{b'}}_{nx}\;$$

(16) $${T\left(x,n\right)}_{Blue}={\vec{b'}}_{ny}\;$$

#### 3.2.2. Gait Classification

The goal of this section is to propose example classification methods that utilize the newly developed gait representations as training and testing data in order to demonstrate their potential. These methods are deliberately kept simple and efficient to enable clear comparisons across different representations. Transfer learning is a widely utilized technique in deep learning that enables the adaptation of a pre-trained network, originally developed for one task, to another relevant task. This approach leverages the knowledge embedded in a network trained on a large, diverse dataset to improve performance on a target task with limited data, a common scenario in biomedical engineering, including gait classification.

A standard implementation of transfer learning involves using a pre-trained network as a feature extractor, processing input data up to its last learnable layer and treating the resulting activations as highly informative features. For example, AlexNet, a pioneering deep convolutional neural network originally trained on ImageNet dataset for image classification, can be repurposed for feature extraction. In this study, we utilized the original 2012 version of AlexNet. This version represented a substantial advancement in deep learning by markedly surpassing prior methodologies in the ImageNet Large Scale Visual Recognition Challenge (ILSVRC 2012). AlexNet consists of five convolutional layers followed by three fully connected layers, with each layer progressively learning to capture more abstract and complex features from the input data. It accepts RGB images of size 224 × 224 × 3 as input and incorporates key design elements such as overlapping max-pooling, local response normalization (LRN), and rectified linear units (ReLU) activation functions, which accelerate convergence and enhance model performance.

To use AlexNet as a feature extractor, the network’s architecture is preserved up to the last learnable layer, typically the second fully connected layer (fc7), before the final classification layer (fc8). The pre-trained weights from the ImageNet training are retained, as they encapsulate general patterns and features learned from a large and diverse dataset. During feature extraction, input images are passed through AlexNet, and the activations from the fc7 layer are extracted. This provides a total of 4096 features. These activations represent a compact and semantically rich feature vector for each input image, encoding high-level visual attributes such as shapes, textures, and patterns. These features are then used as input to a new model or classifier tailored to the target task.

The use of AlexNet for transfer learning offers several advantages. AlexNet’s architecture is relatively simple yet highly effective, making it computationally efficient compared to more recent, deeper models. Its design includes ReLU for non-linearity and dropout for regularization, which contribute to robust learning. Leveraging a pretrained AlexNet eliminates the need to train a deep network from scratch, which is particularly advantageous when working with limited data as mentioned above. Training a deep learning model from scratch is computationally expensive, time-consuming, and prone to overfitting, especially when the dataset is small. By using a pretrained AlexNet, the extracted features capture the generalizable knowledge of the model, ensuring a robust starting point for further analysis. It is important to note that in the proposed framework, AlexNet is not used as an end-to-end classifier but solely as a feature extractor within the transfer learning pipeline. The proposed approach is architecture-agnostic and can easily integrate other pre-trained deep networks. This design choice ensures that the novelty and generalizability of the proposed method stem from the newly developed gait representations and the feature optimization strategy, rather than the specific choice of backbone network. Consequently, if AlexNet were to produce suboptimal features, it could be replaced without affecting the overall framework structure or performance evaluation pipeline.

Since the input to AlexNet is a color image, the network training takes the color information into account resulting in varying processing weights for each of the color channels. In the proposed gait representations, three techniques utilize color coding to encode different types of information. Consequently, the feature extraction process depends on how this information is assigned to the color channels in the input image. In other words, for the same underlying data, altering the order of the color channels will produce different sets of features from AlexNet. To ensure that the most comprehensive set of features is captured, all possible permutations of the color channels (i.e., color shift) are generated and used as inputs to AlexNet, and the resulting feature sets are concatenated. For grayscale representations, this permutation will result in the same set of features repeated in the feature vector, which does not affect the outcome. Given that there are three color channels (R, G, B), the total number of possible permutations is 3!=3×2×1=6 , comprising (R, G, B), (R, B, G), (G, R, B), (G, B, R), (B, R, G), and (B, G, R). Accordingly, for each input image, a feature vector F consisting of 4096×6=24,576 features is obtained. This results in a high-dimensional feature space, which necessitates the application of dimensionality reduction techniques such as principal component analysis (PCA).

PCA is a widely used dimensionality reduction technique which transforms high-dimensional data into a lower-dimensional space while preserving as much variance as possible. It identifies new axes, called principal components, which are linear combinations of the original variables. These components are ordered by the amount of variance they explain, with the first principal component capturing the largest variance and each subsequent component capturing the maximum remaining variance under the constraint of orthogonality to the previous components.

The PCA process involves several key steps. First, the data is centered by subtracting the mean of each feature. Next, the covariance matrix of the data is computed to capture relationships between features. An eigenvalue decomposition or singular value decomposition (SVD) is then performed on the covariance matrix to obtain the eigenvectors and eigenvalues. The eigenvectors represent the directions of the principal components, while the eigenvalues indicate the amount of variance explained by each principal component. The output of PCA includes the eigenvectors matrix (Loading Matrix) that contains the eigenvectors as its columns. Each eigenvector represents a principal component and defines the direction of maximum variance in the data. The entries of the eigenvectors indicate the contributions (or loadings) of the original variables to each principal component. This matrix provides insights into how the original variables are related to the principal components. By retaining only the first few principal components, PCA reduces the dimensionality of the data while preserving its most significant patterns and discarding noise or less informative dimensions. In this study, the process of dimensionality reduction using PCA was performed as follows. Given the multiclass nature of the problem, the process is applied on each class independently to obtain a set of P eigenvectors, where P is arbitrarily selected as 15 to retain most of the variance (at least 80%) in all classes, while providing significant dimensionality reduction. The eigenvectors from all classes are combined to provide a set of N·P eigenvectors. This combined eigenvectors matrix E has dimensionality of $\left(N\cdot P\times 24,576\right)$ (the number of concatenated features is 24,576 in this study). The reduced feature vector R of dimensionality $\left(N\cdot P\times 1\right)$ is then calculated as, $R=E^{T}\cdot F$ . For a 6-class problem and a selected number of retained eigenvectors P of 15, this results in a modest (90×1) reduced feature vector, which is much simpler to deal with for training robust classification models.

The next step is to use lower-complexity conventional machine learning models to perform classification on the extracted reduced set of features. These models are computationally less intensive and require fewer parameters than a fully trained deep network. This significantly reduces the risk of overfitting, as the classifier focuses on task-specific decision boundaries while leveraging the rich, pre-learned representations from AlexNet. The combination of deep feature extraction and simpler classifiers balances performance with computational efficiency, making this approach particularly suitable for practical biomedical engineering problem scenarios where computational resources are limited and datasets are small. Furthermore, this strategy enhances model interpretability, as the contributions of individual features can often be analyzed more easily in conventional models compared to end-to-end deep learning systems.

An effective approach for classification using the extracted features is to employ a K-Nearest Neighbors (KNN) algorithm. KNN is a simple yet powerful non-parametric machine learning model that classifies data points based on the majority class of their nearest neighbors in the feature space. The advantages of KNN over other conventional models lie in its simplicity, interpretability, and absence of assumptions about the underlying data distribution. KNN does not require model training beyond the initial hyperparameter optimization, making it computationally efficient. Instead, it stores the feature vectors and their corresponding labels and performs classification during inference by comparing the distances between feature vectors. This characteristic makes KNN particularly suitable for transfer learning applications, where the extracted features from AlexNet are already highly discriminative and well-structured for distance-based classification. Another significant advantage of using KNN is its flexibility in handling multi-class problems without the need for complex modifications, as it naturally extends to multi-class settings by considering the nearest neighbors’ majority votes. Additionally, KNN’s simplicity reduces the risk of overfitting compared to more complex machine learning models. It benefits from the high-quality feature representations produced by AlexNet, allowing the classifier to focus on exploiting these discriminative features rather than learning complex decision boundaries that may not generalize well. Furthermore, the interpretability of KNN enhances transparency, as predictions can be traced back to specific training examples in the feature space, making it easier to analyze the classification results.

**Figure 9:**
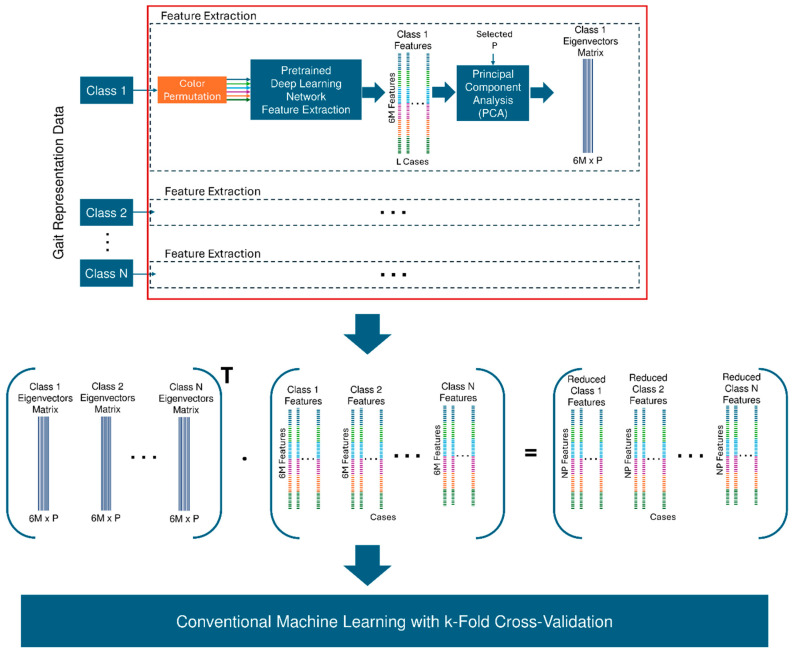
The proposed feature extraction and classification methodology.

In order to ensure reliable estimation of performance, K-fold cross-validation, a robust technique commonly used in machine learning and statistical modeling to evaluate the performance and generalizability of a model, is utilized. It works by partitioning the dataset into K equal (or nearly equal) subsets (or folds). The model is trained on (K − 1) folds and validated on the remaining fold, ensuring that every data point is used for both training and validation exactly once. This process is repeated K times, with a different fold serving as the validation set in each iteration. The results from all K iterations are then averaged to produce a single set of performance metrics, such as accuracy, sensitivity, etc. This method reduces the variability associated with a single train-test split, providing a more reliable estimate of the model’s performance on unseen data. Additionally, K-fold cross-validation helps to detect overfitting and ensures the robustness of the model, making it an essential component of model evaluation in machine learning workflows. For this study, the value of K was selected as 5 to balance computational efficiency with performance reliability. A block diagram of the introduced classification methodology is shown in Figure 9.

## 4. Results and Discussion

In this study, five gait representation maps were compared: the traditional GEI and four newly introduced maps as described above. AlexNet was used for feature extraction along with several conventional machine learning classifiers. Two classification problems were investigated to assess the robustness of the proposed methods under varying complexities: a 4-class task (FB, LL, RL, NM) and a more challenging 6-class task (FB, LL, RL, NM, LA, RA). The following performance metrics were used to assess the maps and algorithms: overall accuracy (Acc), True Positive Rate (TPR), also known as sensitivity (SE) or recall, Area Under the receiver operating characteristic (ROC) Curve (AUC), F1-score (the harmonic mean of precision and recall), and weighted average Positive Predictive Value (PPV). The number of samples for each class was FB (112), LL (102), RL (113), NM (85), LA (90), and RA (85), respectively.

Table 1 summarizes the overall accuracy, weighted-average sensitivity, F1-scores, and AUC achieved across different representations and setups. Without applying color shifts, the GEI map achieved the best performance in the 4-class experiment, with an accuracy of 95.4% and an AUC of 0.994. However, when color shifts were applied, all the proposed representation maps outperformed GEI. In contrast, the cGEI map showed superior performance in the 6-class experiment, with an accuracy of 87.6% and an AUC of 0.983.

**Figure 10:**
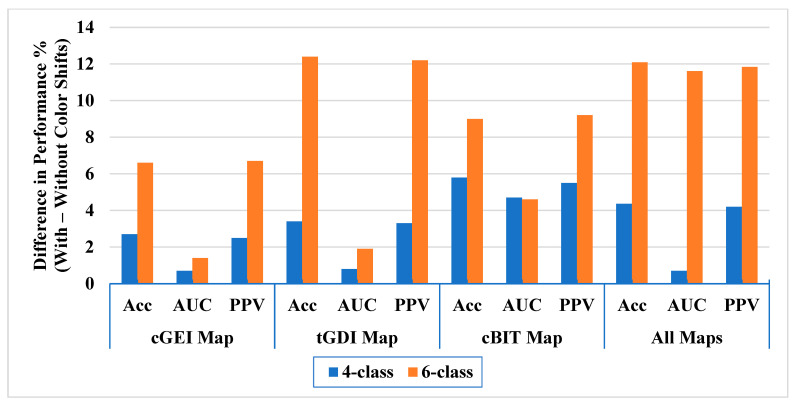
Differences in overall performance with and without the application of color shifts.

Additionally, applying the color shift method improved performance across all experiments and all maps, as well as for the combined features from all maps, as shown in Figure 10. For the 4-class experiment, results improved by 2.5–5.8% in accuracy, 0.7–4.7% in AUC, and 2.5–5.5% in PPV. This demonstrates the substantial benefit of leveraging color permutations (i.e., color shifts) to extract richer feature sets from the representations. The cBIT map exhibited the largest performance gain from color shifts.

For the 6-class experiment, results improved by 6.6–12.4% in accuracy, 1.4–11.6% in AUC, and 6.7–12.2% in PPV. Notably, the gains were more pronounced in this higher complexity task, showing that multi-class problems benefit even more from enriched feature extraction. Classification using the combined features from all maps was maximally enhanced by applying color shifts, suggesting that integrating diverse representations coupled with channel re-encoding offers a powerful approach for robust gait impairment recognition.

**Figure 11:**
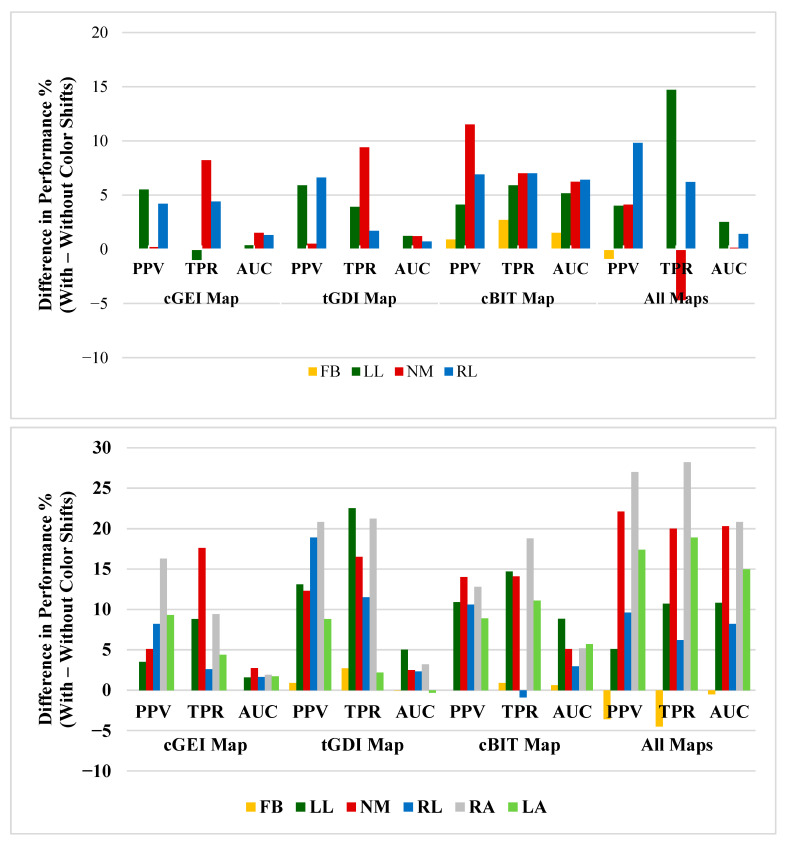
Differences in class-level performance with and without applying color shifts. (**Top**): results for the 4-class experiment; (**bottom**): results for the 6-class experiment.

In the 4-class problem, using the cGEI map resulted in a 1% decrease in the TPR of LL, and combining all features led to a decrease of 0.9% in the PPV of FB and a 4.7% reduction in the TPR of NM. Additionally, in the 6-class problem, using the tGDI map lowered the AUC of LA by 0.31%, the cBIT map reduced the TPR of RL by 0.9%, and combining all features decreased the PPV, TPR, and AUC of FB by 3.6%, 4.5%, and 0.5%, respectively. These relatively minor reductions are likely attributed to inter-class confusion where subtle impairments (e.g., left vs. right arm restrictions) share overlapping gait features, particularly when combined into high-dimensional feature spaces. This is illustrated in Figure 11. Detailed class-level performance with and without the color shift method is shown in Table 2 and Table 3. The FB class consistently achieved the highest values across all metrics in both the 4-class and 6-class experiments. Overall, performance improved in all setups, with the exception of a few specific cases.

Beyond our experiments, these findings connect well with prior research. The GEI map has been widely used and investigated for both gait recognition [6,12,13,14,15,16,17] and anomaly detection tasks [2,3,10,11,28,37]. However, changes in walking speed, clothing, or wearing additional items such as a coats or shoes can significantly degrade the performance of GEI [7,10,13,14,15,16,27,38]. In 2019, Xu et al. [16] introduced the Single Support GEI (SSGEI), a new map designed to be speed-invariant for subject recognition. In our study, we introduce four silhouette-derived feature maps, specifically aimed at classifying gait anomalies.

Earlier, in 2014, Whytock et al. [38] introduced the Skeleton Variance Image (SVIM). Through mathematical manipulation, they also derived the Skeleton Energy Image (SEIM) and Gait Variance Image (GVI) from SVIM and GEI, respectively. In their experiments, GEI performed best under normal conditions (i.e., unchanged clothing and posture). However, its performance dropped markedly when these conditions varied.

In 2020, Loureiro et al. [10] proposed the Skeleton Energy Image (SEI) and created their own dataset, GAIT-IST, consisting of five classes. In their work, they used VGG-19 for feature extraction, and either fed these features directly into VGG-19 for classification or applied PCA followed by LDA or SVM. Under the best configuration, SVM and LDA achieved similar accuracies of 96.4%, whereas using VGG-19 for the entire pipeline reached 98.5%. However, this performance dropped sharply due to overfitting when tested on a different dataset, with the highest accuracy only reaching 76.7%.

Compared to these studies, our proposed framework demonstrated strong generalization within the controlled INIT dataset, especially when leveraging multi-representation combinations and color shifts. Table 4 lists studies that employed GEI or proposed new representation maps for gait anomaly detection. These outcomes suggest that integrating novel representation maps capturing boundary, temporal, and motion-difference features, combined with multi-channel exploitation, provides a promising direction for enhancing automated gait impairment analysis.

While this study primarily utilized silhouettes from the INIT Gait Database, which are acquired under controlled imaging conditions, this choice was deliberate to isolate and evaluate the representational power of the proposed model independently of segmentation and background noise effects. In real-world clinical environments, silhouette extraction is influenced by factors such as background complexity, illumination variation, clothing, and occlusions. These factors constitute a separate preprocessing challenge that directly affects all silhouette-based representations, including GEI. Our contribution, therefore, focuses on improving the robustness and discriminative capability of the gait representation given a reasonably accurate silhouette extraction. Once integrated with advanced segmentation techniques (e.g., background subtraction based on deep learning, adaptive thresholding, or multimodal sensing), the proposed representations can be expected to generalize effectively to clinical settings.

## 5. Conclusions

This study introduces four novel gait representation maps (tGBI, cGEI, tGDI, and cBIT) designed to address the limitations of traditional Gait Energy Images (GEI) by incorporating boundary information, temporal dynamics, and enhanced spatial features. Comprehensive experiments conducted on the INIT Gait dataset demonstrated that the proposed representations—particularly with the application of color shifts—consistently outperform the conventional GEI in classifying gait impairments across multiple machine learning models and varying impairment categories (n = 4 and n = 6 classes). These findings highlight the potential of the proposed methods to improve the accuracy and robustness of automated clinical gait analysis, offering valuable tools for supporting objective and reliable diagnosis in healthcare settings.

## Figures and Tables

**Table 1:** Classification performance of gait impairments using different gait representations.

Map	4-Class Problem		6-Class Problem	
	Without Color Shift	with Color Shift	Without Color Shift	with Color Shift
GEI	Discriminant Analysis	Not Applicable	KNN	Not Applicable
	Acc: 95.4%		Acc: 83.5%	
	AUC: 99.4%		AUC: 98.0%	
	SE: 95.38%		SE: 83.48%	
	F1-score: 95.47%		F1-score: 83.98%	
tGBI	Discriminant Analysis	Not Applicable	KNN	Not Applicable
	Acc: 91.5%		Acc: 83.0%	
	AUC: 98.8%		AUC: 98.0%	
	SE: 91.53%		SE: 82.97%	
	F1-score: 91.64%		F1-score: 83.25%	
cGEI	Discriminant Analysis	KNN	KNN	KNN
	Acc: 94.4%	Acc: 97.1%	Acc: 87.6%	Acc: 94.2%
	AUC: 99.1%	AUC: 99.8%	AUC: 98.3%	AUC: 99.7%
	SE: 94.42%	SE: 97.08%	SE: 87.58%	SE: 94.19%
	F1-score: 94.49%	F1-score: 97.1%	F1-score: 87.58%	F1-score: 94.24%
tGDI	Discriminant Analysis	KNN	KNN	KNN
	Acc: 94.9%	Acc: 98.3%	Acc: 82.8%	Acc: 95.2%
	AUC: 99.1%	AUC: 99.9%	AUC: 97.1%	AUC: 99.0%
	SE: 94.92%	SE: 98.29%	SE: 82.8%	SE: 95.24%
	F1-score: 94.94%	F1-score: 98.31%	F1-score: 82.95%	F1-score: 95.29%
cBIT	KNN	KNN	KNN	KNN
	Acc: 91.5%	Acc: 97.3%	Acc: 83.0%	Acc: 92.0%
	AUC: 94.5%	AUC: 99.2%	AUC: 94.8%	AUC: 99.4%
	SE: 91.74%	SE: 97.3%	SE: 82.98%	SE: 92%
	F1-score: 91.78%	F1-score: 97.32%	F1-score: 82.96%	F1-score: 92.06%
All Maps	SVM	KNN	KNN	KNN
	Acc: 92%	Acc: 96.36%	Acc: 77.17%	Acc: 89.26%
	AUC: 99.0%	AUC: 99.7%	AUC: 86.47%	AUC: 98.08%
	SE: 91.99%	SE: 96.36%	SE: 77.18%	SE: 89.25%
	F1-score: 92.03%	F1-score: 96.35%	F1-score: 77.4%	F1-score: 89.35%

**Table 2:** Individual class performance in the 4-class experiment.

Maps	Class	PPV (%)		TPR/SE (%)		AUC (%)	
		Without Color Shift	With Color Shifts	Without Color Shift	With Color Shifts	Without Color Shift	With Color Shifts
GEI	FB	100.00	Not Applicable	100.00	Not Applicable	100.0	Not Applicable
	LL	92.40		95.10		99.2	
	NM	98.60		85.90		98.9	
	RL	91.70		98.20		99.4	
	Average	95.55		95.38		99.4	
tGBI	FB	100.00	Not Applicable	99.10	Not Applicable	99.4	Not Applicable
	LL	89.00		87.30		98.4	
	NM	93.20		81.20		98.1	
	RL	85.00		95.60		99.2	
	Average	91.76		91.53		98.8	
cGEI	FB	100.00	100.00	100.00	100.00	100.0	100.0
	LL	91.40	96.90	94.10	93.10	99.4	99.7
	NM	97.40	97.60	87.10	95.30	98.2	99.7
	RL	89.90	94.10	94.70	99.10	98.5	99.8
	Average	94.56	97.12	94.42	97.08	99.1	99.8
tGDI	FB	100.00	100.00	100.00	100.00	100.0	100.0
	LL	93.10	99.00	92.20	96.10	98.6	99.9
	NM	95.00	95.50	89.40	98.80	98.5	99.7
	RL	91.60	98.20	96.50	98.20	99.1	99.8
	Average	94.96	98.33	94.92	98.29	99.1	99.8
cBIT	FB	99.10	100.00	97.30	100.00	98.5	100.0
	LL	90.90	95.00	88.20	94.10	92.7	97.8
	NM	88.50	100.00	90.60	97.60	93.8	100.0
	RL	87.90	94.80	90.30	97.30	92.8	99.2
	Average	91.81	97.34	91.75	97.30	94.5	99.2
All Maps	FB	100.00	99.10	100.00	100.00	100.0	100.0
	LL	91.10	95.10	80.40	95.10	97.3	99.9
	NM	92.30	96.40	98.80	94.10	99.6	99.7
	RL	84.90	94.70	89.40	95.60	97.9	99.3
	Average	92.07	96.35	91.99	96.36	98.7	99.7

**Table 3:** Individual class performance in the 6-class experiment.

Maps	Class	PPV (%)		TPR/SE (%)		AUC (%)	
		Without Color Shift	With Color Shifts	Without Color Shift	With Color Shifts	Without Color Shift	With Color Shifts
GEI	FB	100.00	Not Applicable	100.00	Not Applicable	100.0	Not Applicable
	LA	76.70		76.70		96.1	
	LL	95.50		83.30		98.7	
	NM	74.30		64.70		95.4	
	RA	61.50		78.80		95.7	
	RL	90.30		90.30		98.5	
	Average	84.48		83.48		97.6	
tGBI	FB	100.00	Not Applicable	100.00	Not Applicable	100.0	Not Applicable
	LA	73.20		78.90		96.8	
	LL	94.00		77.50		97.5	
	NM	77.30		68.20		95.7	
	RA	72.00		78.80		96.7	
	RL	79.40		88.50		98.3	
	Average	83.54		82.97		97.6	
cGEI	FB	100.00	100.00	100.00	100.00	100.0	100.0
	LA	85.10	94.40	88.90	93.30	97.8	99.5
	LL	90.70	94.20	86.30	95.10	98.2	99.8
	NM	80.30	85.40	71.80	89.40	96.3	99.1
	RA	78.90	95.20	83.50	92.90	97.9	99.8
	RL	86.40	94.60	90.30	92.90	97.9	99.6
	Average	87.57	94.29	87.58	94.19	98.1	99.6
tGDI	FB	99.10	100.00	97.30	100.00	100.0	100.0
	LA	82.10	90.90	86.70	88.90	97.6	97.3
	LL	85.90	99.00	71.60	94.10	94.2	99.2
	NM	77.60	89.90	77.60	94.10	96.8	99.3
	RA	70.30	91.10	75.30	96.50	95.0	98.2
	RL	79.30	98.20	85.00	96.50	97.0	99.4
	Average	83.11	95.33	82.80	95.24	96.9	99.0
cBIT	FB	100.00	100.00	99.10	100.00	99.4	100.0
	LA	80.60	89.50	83.30	94.40	93.9	99.6
	LL	82.10	93.00	76.50	91.20	90.4	99.3
	NM	77.20	91.20	71.80	85.90	93.5	98.6
	RA	69.80	82.60	70.60	89.40	93.4	98.6
	RL	82.90	93.50	90.30	89.40	96.5	99.5
	Average	82.95	92.13	82.98	92.0	94.7	99.3
All Maps	FB	100.00	96.40	100.00	95.50	100.0	99.5
	LA	66.30	83.70	72.20	91.10	82.8	97.8
	LL	84.90	90.00	77.50	88.20	87.3	98.1
	NM	63.60	85.70	57.60	77.60	76.0	96.3
	RA	55.80	82.80	62.40	90.60	77.0	97.8
	RL	84.80	94.40	84.10	90.30	90.2	98.4
	Average	77.61	89.44	77.18	89.25	86.5	98.1

**Table 4:** Summary of recent studies using GEI-based or novel gait maps for anomaly detection and their best reported performance.

Reference	Map/Feature Extraction Approach	Classifier	Dataset(s)	Classes	Performance
Verlekar et al., 2018 [2]	GEI and silhouette features using image processing.	SVM	INIT	FB, LL, RL, and NM	Acc.: 98.8%
Elkholy et al., 2019 [37]	GEI and applying convolutional autoencoder	Isolation forests	OU-ISIR (train) [39] and INIT (test)	Normal and abnormal	AUC: 0.94
Gong et al., 2020 [11]	GEI and applying R-CNN on video clips	OC-SVM	Their own dataset and Youtube videos	Normal and Parkinsonian	Acc. 97.33%
Loureiro et al., 2020 [10]	GEI and SEI (VGG-19)	VGG-19	GAIT-IST	Normal, diplegic, hemiplegic, neuropathic, and Parkinsonian	Acc.: 98.5%
	GEI and SEI (VGG-19 + PCA)	LDA / SVM	GAIT-IST		Acc.: 96.4%
	GEI (VGG-19 + PCA)	LDA	GAIT-IST (train) and DAI 2 (test) [40]		Acc.: 76.7%
Zhou et al. 2024 [3]	GEI (lightweight CNN)	CNN	GAIT-IST [10]	Normal, diplegic, hemiplegic, neuropathic and Parkinsonian	Acc.: 98.1%
	GEI (lightweight CNN)	CNN	GAIT-IST	Normal + 3 levels hemiplegia	Acc.: 96.92%
Al-masni et al., 2024 [28]	GEI (2D CNN)	CNN	INIT	FB, LL, RL, LA, RA, and NM	Acc. 70.94%
	GEI (ResNet50)	ResNet50		FB, LL, RL, and NM	Acc.: 86.25%
**Proposed Method**	New maps (AlexNet features)	KNN	INIT	FB, LL, RL, and NM	Acc.: 98.3%
					F1 score: 98.31%
					AUC: 0.999
				FB, LL, RL, LA, RA, and NM	Acc.: 95.2%
					F1 score: 95.29%
					AUC: 0.990

## Data Availability

The authors confirm that the data used in this study are publicly available at https://www.vision.uji.es/gaitDB/ (accessed on 5 March 2025).

## References

[1] Matsushita Y., Tran D.T., Yamazoe H., Lee J.-H. (2021). Recent use of deep learning techniques in clinical applications based on gait: A survey. J. Comput. Des. Eng..

[2] Verlekar T.T., Soares L.D., Correia P.L. (2018). Automatic classification of gait impairments using a markerless 2D video-based system. Sensors.

[3] Zhou C., Feng D., Chen S., Ban N., Pan J. (2024). Portable vision-based gait assessment for post-stroke rehabilitation using an attention-based lightweight CNN. Expert Syst. Appl..

[4] Ranjan R., Ahmedt-Aristizabal D., Armin M.A., Kim J. (2024). Computer Vision for Clinical Gait Analysis: A Gait Abnormality Video Dataset. arXiv.

[5] Whittle M.W. (2014). Gait Analysis: An introduction.

[6] Song X., Hou S., Huang Y., Cao C., Liu X., Huang Y., Shan C. (2023). Gait Attribute Recognition: A New Benchmark for Learning Richer Attributes from Human Gait Patterns. IEEE Trans. Inf. Forensics Secur..

[7] Wu L.C., Kuo C., Loza J., Kurt M., Laksari K., Yanez L.Z., Senif D., Anderson S.C., Miller L.E., Urban J.E. (2017). Detection of American football head impacts using biomechanical features and support vector machine classification. Sci. Rep..

[8] El-Ziaat H., El-Bendary N., Moawad R. Using Multi-Feature Fusion for Detecting Freezing of Gait Episodes in Patients with Parkinson’s Disease. Proceedings of the 2020 International Conference on Innovative Trends in Communication and Computer Engineering (ITCE).

[9] Lin C.-W., Wen T.-C., Setiawan F. (2020). Evaluation of vertical ground reaction forces pattern visualization in neurodegenerative diseases identification using deep learning and recurrence plot image feature extraction. Sensors.

[10] Loureiro J., Correia P.L. Using a skeleton gait energy image for pathological gait classification. Proceedings of the 2020 15th IEEE International Conference on Automatic Face and Gesture Recognition (FG 2020).

[11] Gong L., Li J., Yu M., Zhu M., Clifford R. A novel computer vision based gait analysis technique for normal and Parkinson’s gaits classification. Proceedings of the 2020 IEEE Intl Conf on Dependable, Autonomic and Secure Computing, Intl Conf on Pervasive Intelligence and Computing, Intl Conf on Cloud and Big Data Computing, Intl Conf on Cyber Science and Technology Congress (DASC/PiCom/CBDCom/CyberSciTech).

[12] Han J., Bhanu B. (2005). Individual recognition using gait energy image. IEEE Trans. Pattern Anal. Mach. Intell..

[13] Gupta S.K., Chattopadhyay P. (2021). Exploiting pose dynamics for human recognition from their gait signatures. Multimed. Tools Appl..

[14] Bukhari M., Bajwa K.B., Gillani S., Maqsood M., Durrani M.Y., Mehmood I., Ugail H., Rho S. (2020). An efficient gait recognition method for known and unknown covariate conditions. IEEE Access.

[15] Lenac K., Sušanj D., Ramakić A., Pinčić D. (2019). Extending appearance based gait recognition with depth data. Appl. Sci..

[16] Xu C., Makihara Y., Li X., Yagi Y., Lu J. (2019). Speed-invariant gait recognition using single-support gait energy image. Multimed. Tools Appl..

[17] Yao L., Kusakunniran W., Wu Q., Zhang J., Tang Z., Yang W. (2021). Robust gait recognition using hybrid descriptors based on skeleton gait energy image. Pattern Recognit. Lett..

[18] Ahlrichs C., Samà A., Lawo M., Cabestany J., Rodríguez-Martín D., Pérez-López C., Sweeney D., Quinlan L.R., Laighin G.Ò., Counihan T. (2016). Detecting freezing of gait with a tri-axial accelerometer in Parkinson’s disease patients. Med. Biol. Eng. Comput..

[19] Saad A., Zaarour I., Guerin F., Bejjani P., Ayache M., Lefebvre D. (2017). Detection of freezing of gait for Parkinson’s disease patients with multi-sensor device and Gaussian neural networks. Int. J. Mach. Learn. Cybern..

[20] Linda G.M., Themozhi G., Bandi S.R. (2020). Color-mapped contour gait image for cross-view gait recognition using deep convolutional neural network. Int. J. Wavelets Multiresolution Inf. Process..

[21] Sharma O., Bansal S. (2013). Gait Recogniton System for Human Identification Using BPNN Classifier. Int. J. Innov. Technol. Explor. Eng..

[22] Zhang C., Liu W., Ma H., Fu H. Siamese neural network based gait recognition for human identification. Proceedings of the 2016 IEEE International Conference on Acoustics, Speech and Signal Processing (ICASSP).

[23] Gaba I., Ahuja S.P. Gait analysis for identification by using BPNN with LDA and MDA techniques. Proceedings of the 2014 IEEE International Conference on MOOC, Innovation and Technology in Education (MITE).

[24] Castro F.M., Marín-Jiménez M.J., Guil N., Perez De La Blanca N. (2017). Automatic learning of gait signatures for people identification. Proceedings of the International Work-Conference on Artificial Neural Networks.

[25] Hawas A.R., El-Khobby H.A., Abd-Elnaby M., Abd El-Samie F.E. (2019). Gait identification by convolutional neural networks and optical flow. Multimed. Tools Appl..

[26] Alotaibi M., Mahmood A. (2017). Improved gait recognition based on specialized deep convolutional neural network. Comput. Vis. Image Underst..

[27] Nahar S., Narsingani S., Patel Y. (2023). Cross View and Cross Walking Gait Recognition Using a Convolutional Neural Network. Proceedings of the International Conference on Computer Vision and Image Processing.

[28] Al-Masni M.A., Marzban E.N., Al-Shamiri A.K., Al-Antari M.A., Alabdulhafith M.I., Mahmoud N.F., Abdel Samee N., Kadah Y.M. (2024). Gait Impairment Analysis Using Silhouette Sinogram Signals and Assisted Knowledge Learning. Bioengineering.

[29] Gul S., Malik M.I., Khan G.M., Shafait F. (2021). Multi-view gait recognition system using spatio-temporal features and deep learning. Expert Syst. Appl..

[30] Mogan J.N., Lee C.P., Lim K.M., Ali M., Alqahtani A. (2023). Gait-CNN-ViT: Multi-model gait recognition with convolutional neural networks and vision transformer. Sensors.

[31] Yunardi R.T., Sardjono T.A., Mardiyanto R. (2024). Skeleton-Based Gait Recognition Using Modified Deep Convolutional Neural Networks and Long Short-Term Memory for Person Recognition. IEEE Access.

[32] Ghosh R. (2022). A Faster R-CNN and recurrent neural network based approach of gait recognition with and without carried objects. Expert Syst. Appl..

[33] Junaid M.I., Prakash A.J., Ari S. (2024). Human gait recognition using joint spatiotemporal modulation in deep convolutional neural networks. J. Vis. Commun. Image Represent..

[34] Nahar S., Narsingani S., Patel Y. (2023). A Unified Convolutional Neural Network for Gait Recognition. Proceedings of the Asian Conference on Pattern Recognition.

[35] Song C., Huang Y., Huang Y., Jia N., Wang L. (2019). Gaitnet: An end-to-end network for gait based human identification. Pattern Recognit..

[36] Gonzalez R.C. (2009). Digital Image Processing.

[37] Elkholy A., Makihara Y., Gomaa W., Ahad M.A.R., Yagi Y. Unsupervised GEI-based gait disorders detection from different views. Proceedings of the 2019 41st Annual International Conference of the IEEE Engineering in Medicine and Biology Society (EMBC).

[38] Whytock T., Belyaev A., Robertson N.M. (2014). Dynamic distance-based shape features for gait recognition. J. Math. Imaging Vis..

[39] Iwama H., Okumura M., Makihara Y., Yagi Y. (2012). The OU-ISIR gait database comprising the large population dataset and performance evaluation of gait recognition. IEEE Trans. Inf. Forensics Secur..

[40] Nieto-Hidalgo M., García-Chamizo J.M. Classification of pathologies using a vision based feature extraction. Proceedings of the Ubiquitous Computing and Ambient Intelligence: 11th International Conference, UCAmI 2017.

